# Supplementary material for: Independent assessment of a point of care HCV RNA test by laboratory analytical testing and a prospective field study in the U.S
Source: PLoS One. 2025 Jul 22;20(7):e0324088. doi: 10.1371/journal.pone.0324088 (PMC12282913; doi:10.1371/journal.pone.0324088)
Supplement: Supplementary Table 1 — (DOCX) [file pone.0324088.s001.docx]

**Supporting Information**

**Supplementary Table 1. Quantitation of HCV Genotype 1a clinical samples used for CWB spiking in sample stability study.**

| Patient ID (Anonymized) | Month Collected | Matrix | Cobas^®^ HCV Quantitation (IU/ml) |
| --- | --- | --- | --- |
| 001 | September 2023 | Serum | 530,200 |
| V002 | November 2023 | Plasma | 1,892,000 |
| 003 | December 2023 | Plasma | 2,842,000 |
| 004 | December 2023 | Plasma | 66,980 |
| 005 | November 2023 | Plasma | 389,800 |

All samples were quantified using Cobas 6800. The IU/ml values obtained from cobas 6800 were used to calculate the amount of material to spike into CWB -microtainer stability studies. IU/ml, International Units per milliliter; CWB, Capillary whole Blood.
