# Supplementary material for: Independent assessment of a point of care HCV RNA test by laboratory analytical testing and a prospective field study in the U.S
Source: PLoS One. 2025 Jul 22;20(7):e0324088. doi: 10.1371/journal.pone.0324088 (PMC12282913; doi:10.1371/journal.pone.0324088)
Supplement: Supplementary Table 2 — (DOCX) [file pone.0324088.s002.docx]

**Supplementary Table 2. Quantitation, and source for HCV Genotypes used in analytical Limit of Detection (LOD) of HCV Genotypes 1-6 in CWB and VWB.**

| Genotype | Sample Type | Stock Concentration (IU/ml) | Catalog Number | Source |
| --- | --- | --- | --- | --- |
| Genotype 1a | Human source material in lyophilized plasma, reconstitute in water | 233636 IU/ml (COA) | 18/184 | NIBSC (https://nibsc.org) |
| Genotype 1b | HCV positive serum | 3,908,000 IU/ml (as determined by cobas^®^ HCV | DLS16-72210 | Discovery LifeSciences (https://dls.com) |
| Genotype 2b | Serum SST | 1074446 IU/ml as determined by cobas^®^ HCV | 177102-13 | Biocollections Worldwide (https://www.biocollections.com) |
| Genotype 3a | Plasma | 11,180,000 IU/ml as determined by cobas^®^ HCV | 141199-66 | Biocollections Worldwide |
| Genotype 4 | Plasma | 629,400 IU/ml as determined cobas^®^ HCV | 184895-13 | Biocollections Worldwide |
| Genotype 5 (used at Emory) | Plasma | 634400 IU/ml as determined by cobas^®^ HCV | 0HC0010100240009068 | FIND |
| Genotype 5 (used at MRI) | Plasma | 449000 IU/ml as determined by cobas^®^ HCV | OHC0010100240009071 | FIND |
| Genotype 6 | Plasma | 495,800 IU/ml as determined cobas^®^ HCV | DLS16-67657 | Discovery Life Science |

HCV genotypes used for this study were purchased from commercial sources. The concentration of genotype 1a was denoted in the COA. All other concentrations were determined using the cobas 6800. COA, Certificate of Analysis.
