# Supplementary material for: Independent assessment of a point of care HCV RNA test by laboratory analytical testing and a prospective field study in the U.S
Source: PLoS One. 2025 Jul 22;20(7):e0324088. doi: 10.1371/journal.pone.0324088 (PMC12282913; doi:10.1371/journal.pone.0324088)
Supplement: Supplementary Table 3 — (DOCX) [file pone.0324088.s003.docx]

**Analytical Limit of Detection (LOD) of HCV Genotypes 1-6 in CWB and VWB.**

All HCV genotypes were quantitated using the cobas^®^ HCV test to verify starting concentrations used for each experiment, and these values were used for all spiking experiments. All donors who contributed CWB for LOD studies were first screened for HCV by cobas^®^ HCV and only HCV negative donors contributed. All HCV stocks (Supplementary Table 2) were diluted such that they were 10-fold more concentrated than the required final concentration to be tested using HCV negative VWB as matrix. The 10- fold stock was spiked to CWB to prepare 1X HCV. The amount of VWB did not exceed 10% in each test sample. For all genotypes, an initial range finding study was carried out where 3 to 5 dilutions per genotype and 5 replicates per dilution were tested. Each replicate used CWB obtained from a different individual collected in a BD microtainer (**Supplementary Table 3)**. Using a precision pipette, 108μl CWB was transferred to a microtainer tube, to which 12μl 10-fold HCV stock was added for a final concentration of 1X. After mixing by gentle pipetting, 100μl was transferred to the Xpert® HCV test cartridge and tested. Range finding was conducted for all genotypes prior to the LoD confirmation studies. For example, to prepare 100 IU/ml of genotype 1a, we use the stock at a concentration of 233636 IU/ml and dilute to 1000 IU/ml. Then, to prepare 100 IU/ml test sample, 12μl of 1000 IU/ml was spiked into 108μl CWB for 120μl 100 IU/ml, out of which 100μl was tested.

**Supplementary Table 3. Schematic followed for HCV range finding studies.**

| Range Finding (5 replicates) | | | | | |
| --- | --- | --- | --- | --- | --- |
| 100 IU/ml | Person 1 | Person 2 | Person 3 | Person 4 | Person 5 |
| 70 IU/ml | Person 1 | Person 2 | Person 3 | Person 4 | Person 5 |
| 45 IU/ml | Person 1 | Person 2 | Person 3 | Person 4 | Person 5 |
| 37.5 IU/ml | Person 1 | Person 2 | Person 3 | Person 4 | Person 5 |
| 18.75 IU/ml | Person 1 | Person 2 | Person 3 | Person 4 | Person 5 |
| 0 IU/ml | Person 1 | Person 2 | Person 3 | Person 4 | Person 5 |

Five different CWB samples were used for each replicate of every dilution. All concentrations were tested in replicates of five. CWB, capillary whole blood.
