# Supplementary material for: Independent assessment of a point of care HCV RNA test by laboratory analytical testing and a prospective field study in the U.S
Source: PLoS One. 2025 Jul 22;20(7):e0324088. doi: 10.1371/journal.pone.0324088 (PMC12282913; doi:10.1371/journal.pone.0324088)
Supplement: Supplementary Table 4 — (DOCX) [file pone.0324088.s004.docx]

**Supplementary Table 4: Schematic followed for HCV LoD confirmation studies.**

| Initial conc. (IU/ml) | Final conc. (IU/ml) | LOD Confirmation (20 replicates) | | | | | | | | | | | | | | | | | | | |
| --- | --- | --- | --- | --- | --- | --- | --- | --- | --- | --- | --- | --- | --- | --- | --- | --- | --- | --- | --- | --- | --- |
| 1000 | **100** | P1 | P1 | P1 | P1 | P2 | P2 | P2 | P2 | P3 | P3 | P3 | P3 | P4 | P4 | P4 | P4 | P5 | P5 | P5 | P5 |
| 500 | **50** | P1 | P1 | P1 | P1 | P2 | P2 | P2 | P2 | P3 | P3 | P3 | P3 | P4 | P4 | P4 | P4 | P5 | P5 | P5 | P5 |
| 37.5 | **37.5** | P6 | P6 | P6 | P6 | P7 | P7 | P7 | P7 | P8 | P8 | P8 | P8 | P9 | P9 | P9 | P9 | P10 | P10 | P10 | P10 |
| 18.75 | **18.75** | P6 | P6 | P6 | P6 | P7 | P7 | P7 | P7 | P8 | P8 | P8 | P8 | P9 | P9 | P9 | P9 | P10 | P10 | P10 | P10 |

Five different CWB samples were used for every four replicates of every dilution. CWB, capillary whole blood.

Example of the scheme followed all LOD confirmation experiments. Persons (P) 1-5 and 6-10 will donate CWB. We will use 450 μl CWB+50 μl stock to make test samples. This will be enough for 4 replicates and overage for a pipetting margin. We used a minimum of 5 donors per LOD confirmation.

For LOD confirmation studies, 20 replicates were tested for each dilution. The dilutions were selected based on range finding data, and the lowest dilution that resulted in 5/5 positive was used as the starting concentration. For the 20 replicates, CWB from 5 donors were used such that no more than 4 replicates per dilution were from the same donor. The schematic shown in **Supplementary Table 4** was followed. Donor numbers, replicates distribution, and dilutions were recommended by the FDA.

For LoD confirmation, we aimed for one dilution to generate a positive hit rate of 0.60 to 0.90 and one dilution to generate 100% positive results. If one of the initial 3 dilutions did not generate a 0.95 positive hit rate, a fourth dilution was tested. A minimum of three dilutions is necessary for conducting Probit analysis. Analyse-it® for Microsoft® Excel (v 6.15.4) Method Validation edition was used for calculating Probit LoD (positive rate at 0.95 probability, data points aggregated, and output log transformed per developer Statistical Reference Guide). Both range finding and LoD confirmation data were used for Probit analysis. All data generated irrespective of positive percent rate were included in Probit analysis. Data points entered into Probit calculations were identified by their concentrations (IU/mL) and assigned a nominal, binary indicator of either ‘0’ for a negative result or ‘1’ for a positive result. Invalid tests were assigned no indicator and were excluded from analysis.

Studies with VWB were conducted by MRIGlobal (Kansas City, MO). K2 VWB purchased from BioIVT and confirmed negative for HCV by Cobas HCV 5800/6800/8800 Systems was used as matrix. HCV genotypes indicated in Supplementary Table 2 were used to contrive VWB samples tested. All HCV genotypes used for CWB and VWB LoD studies were identical except Genotype 5 as the Foundation for Innovative New Diagnostics (FIND) does not allow transfer of samples between institutions.
