# Supplementary material for: Independent assessment of a point of care HCV RNA test by laboratory analytical testing and a prospective field study in the U.S
Source: PLoS One. 2025 Jul 22;20(7):e0324088. doi: 10.1371/journal.pone.0324088 (PMC12282913; doi:10.1371/journal.pone.0324088)
Supplement: S1 Text — (DOCX) [file pone.0324088.s007.docx]

**Supporting Information**

**Supplementary Statistical Analysis**

Regarding historical HCV RNA results, the percentage of subjects with positive HCV RNA was calculated using a denominator of 37, which represented all subjects with a positive HCV antibody. For all other percentages, a denominator of 89 was used to reflect the number of participants in the final study population. To assess the performance of the Xpert® HCV test compared to cobas^®^ HCV RNA testing, a 2 × 2 table was constructed, and sensitivity and specificity were calculated and rounded to the nearest whole percentage. For all analyses, any study participant who was either missing a result or had an invalid/error/no result for either the Xpert® HCV test or the cobas^®^ HCV test was excluded.
